# Supplementary material for: Post-Polio Syndrome Revisited
Source: Neurol Int. 2023 Apr 13;15(2):569–79. doi: 10.3390/neurolint15020035 (PMC10123742; doi:10.3390/neurolint15020035)
Supplement: Supplementary file 1 [file neurolint-15-00035-s001.zip › neurolint-2257132-supplementary.pdf]

Supplementary Figure S1

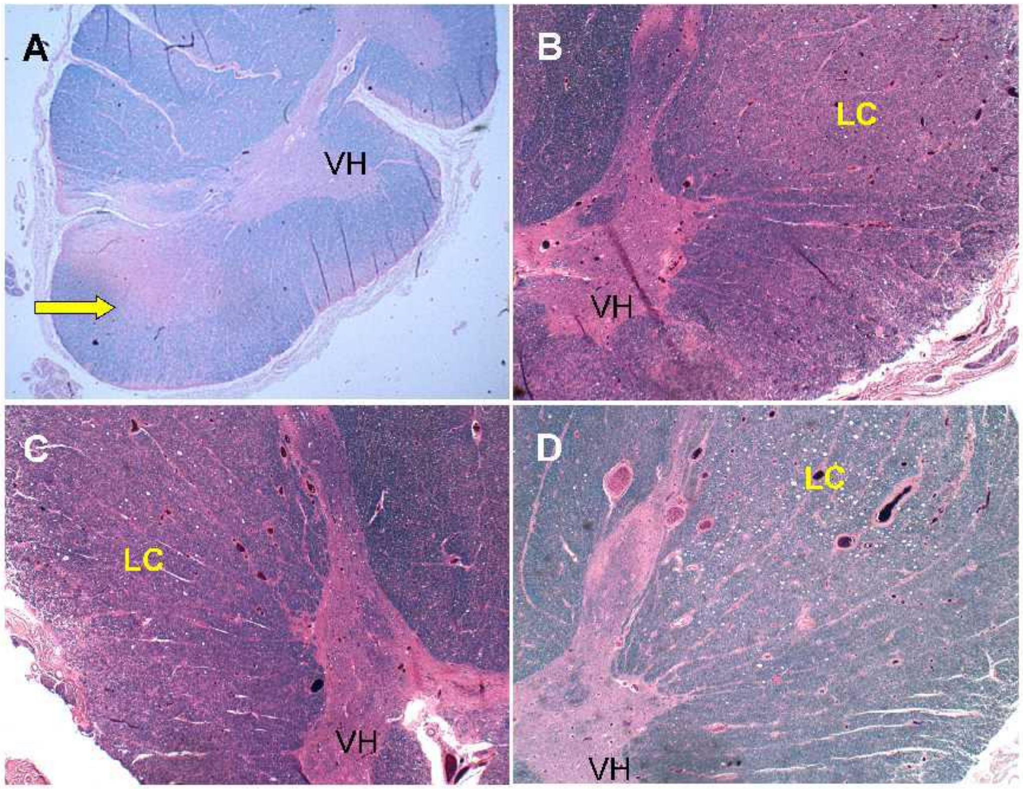

Supplementary Figure S1: Corticospinal tract degeneration in ALS. Formalin fixed, paraffin embedded histological sections of A, B) cervical or C, D) thoracic spinal cord stained with Luxol fast blue, hematoxylin and eosin (LHE) revealed various degrees of tract degeneration in the lateral columns (LC) ranging from A) long established with dense gliosis and myelin pallor, to B, C) moderate but extensive with clear but less well-delineated regions of myelin pallor and vacuolation, to D) mild with subtle myelin pallor but prominent vacuolation. Compare regions of myelin pallor

(pink) with the denser luxol fast blue staining in the posterior columns. Ventral horns (VH) are atrophic, but in Panel B, the degeneration is quite severe, resulting in a blunted appearance.
